# New Advancements in the Field of Leishmaniasis

**DOI:** 10.3390/microorganisms14040845

**Published:** 2026-04-09

**Authors:** Eva Iniguez, Ana Victoria Ibarra-Meneses, Antonio Mastroianni, Isabel L. Mauricio, Pedro Cecilio

**Affiliations:** 1 Vector Molecular Biology Section, Laboratory of Malaria and Vector Research, National Institute of Allergy and Infectious Diseases, National Institutes of Health, Rockville, MD 20852, USA; 2 Département de Pathologie et Microbiologie, Faculté de Médecine Vétérinaire, Université de Montréal, Saint-Hyacinthe, QC J2S 8H5, Canada; 3 Infectious and Tropical Diseases Unit, Azienda Ospedaliera di Cosenza, 87100 Cosenza, Italy; antoniomastroianni@yahoo.it; 4 Global Health and Tropical Medicine (GHTM), Associated Laboratory in Translation and Innovation Towards Global Health (LA-REAL), Instituto de Higiene e Medicina Tropical (IHMT), Universidade NOVA de Lisboa, 1099-085 Lisboa, Portugal; isabel.mauricio@ihmt.unl.pt; 5 Vector Biology Section, Laboratory of Malaria and Vector Research, National Institute of Allergy and Infectious Diseases, National Institutes of Health, Rockville, MD 20852, USA

Leishmaniasis is the term used to characterize a spectrum of human diseases caused by more than 20 different *Leishmania* parasite species [1,2]. Most if not all of these parasites are zoonotic agents, often transmitted to humans accidentally via sand fly bites [1]—more than 90 species, mainly within the *Phlebotomus* and *Lutzomyia* genera, are proven or probable vectors [3]. These aspects highlight the complexity we need to consider when we study leishmaniasis and are one of the justifications for the several limitations associated with the current control tools. No prophylactic or therapeutic vaccine has been approved for human use [4], while the molecules available to treat active cases are only a handful, are often toxic, require long treatment regimens that end up affecting adherence [5], and increasingly fail due to emerging drug resistance [6]. Moreover, the effective control of sand fly vectors as well as the identification and management of animal reservoirs are challenging, particularly considering sylvatic cycles, and often not that successful [7,8]. These are the main reasons why, each year, more than one million leishmaniasis cases (associated with significant mortality and morbidity) are expected to occur, and 350 million people remain at risk of infection worldwide [2,9]. Significant reductions in these numbers are dependent on the advancement of our knowledge on vector–host–parasite interactions for the development of effective and targeted control approaches.

For this Special Issue of *Microorganisms*, entitled “**New Advancements in the Field of Leishmaniasis**”, we invited original contributions describing new developments in the field of leishmaniasis, focusing either on the *Leishmania*–sand fly or *Leishmania*–host interfaces or on the integration of the vector–host–pathogen triad. A total of 65 authors contributed with seven publications: five original research articles, one case report, and one review article.

Dogs are the subject of three of these papers; of note, dogs are the main (peri-)domestic reservoirs of *Leishmania* parasites, particularly *Leishmania infantum* [10,11,12]. The first study focuses on the treatment of *L. infantum* infections in dogs. In an extensive and comprehensive review article based on numerous clinical studies, Kaempfle et al. provide an overview of the molecules currently available to treat canine leishmaniosis (using the dichotomy of leishmanistatic *versus* leishmanicidal drugs), the relevant therapeutic(/prophylactic) schemes, the related adverse effects, and the potential for emergence of drug resistance [10]. First, and wisely, they conclude that any treatment recommendation needs to be based on the individual situation of each dog, including the presence of any comorbidity. This said, they stress that the algorithms that inform the treatment of canine leishmaniosis need to be used transversally and optimized based on scientific data; for this, new studies are needed, designed following standards that allow inter-study comparisons. Such improvements in these algorithms will also contribute to preventing the emergence of drug resistance, whose mechanisms, as the authors state, still need to be better understood.

The second study is pathology-based and looks at an organ not recognized as a target of *Leishmania* parasites. Gonçalves et al. aimed to characterize the expression of fibrosis-related cell markers in the context of chronic interstitial pneumonitis in dogs infected with *L. infantum* parasites, either (i) naturally, or (ii) experimentally, the latter with two different strains [11]. Curiously, in this descriptive study, *L. infantum* amastigotes were microscopically observed in the lungs of 7/11 naturally infected dogs, and in 5/5 dogs experimentally infected with the BH401 *L. infantum* strain, while in none of the seven dogs infected with the BH46 *L. infantum* strain. This said, parasite DNA was detected in the lungs of the remaining three naturally infected dogs and in 4/7 animals infected with *L. infantum* BH46. Notably, parasites were detected in the classical organs targeted by *Leishmania* in all infected animals but not in those of uninfected animals. In line with these data, the authors found extensive pathological changes in the lungs of naturally infected dogs as well as in those experimentally infected with the *L. infantum* BH401 strain, including thickening of the alveolar septa due to chronic exudates of mononuclear cells associated with fibrogenesis, an increased expression of alpha-actin, vimentin, and TGF-β, and a moderate to intense expression of Snail. In contrast, only mild changes were detected in the lungs of dogs experimentally infected with the *L. infantum* BH46 strain, with no changes in the lungs of uninfected animals. Overall, the authors conclude that mesenchymal cells promote changes in the extracellular matrix of the lungs of dogs infected with *L. infantum* to different extents, depending on strain-specific parasite virulence. Further studies are needed to understand the mechanism behind this phenotype and how to prevent its development. Such studies may be relevant not only in the context of canine leishmaniosis, but also of human visceral leishmaniasis (HVL), as there are reports of interstitial pneumonitis in humans with HVL [13,14], and studies showing that alterations in the respiratory system in VL are more common than previously thought [15]. 

The third and last study on dogs focuses on a classic target organ of *Leishmania* parasites, the liver, and, more specifically, on two liver cell types: hepatocytes and Kupffer cells (KCs). Using an in vitro co-culture system, Rodrigues et al. proposed to study hepatocyte–KC interactions and their importance in the context of anti-*Leishmania* liver immune responses [12]. Overall, the authors show that these cells respond differently when in the co-culture system, particularly in terms of cytokine gene expression, e.g., with an upregulation of the mRNA levels of TNF-α, a key cytokine for granuloma formation [16]. This was accompanied by a relevant upregulation of the expression of the regulatory cytokines IL-10 and TGF-β, perhaps a compensatory mechanism that, in the context of the liver, would prevent excessive inflammation and tissue damage [17]. Based on these results, the authors speculate that hepatocytes are the key cells that activate the liver immune responses against *L. infantum* parasites (via interaction with infected KCs), leading to the downstream recruitment of circulating immune cells and the ultimate control of *Leishmania* infection in this organ. This speculation needs, however, to be confirmed, ideally in the context of more complex in vitro systems, e.g., co-culture systems including liver organoids [18] (as the authors assume, their *in vitro* system is simple, only allowing to investigate close cell-to-cell communication).

Two other studies focus on sand fly vectors, their gut microbiota, and their interaction with *Leishmania* parasites [19,20]. The first study by Tempone et al. focuses on *Nyssomyia umbratilis* sand flies, vectors of *Leishmania* (*Viannia*) *guyanensis*-caused American Tegumentary Leishmaniasis (ATL) in the Brazilian Amazon. More specifically, the authors proposed to dissect the differences in the (meta)proteomes of two sand fly populations collected from nearby municipalities with contrasting ATL incidence: Rio Preto da Eva (RPE; high incidence) and Manacapuru (MAN; low incidence) [19]. The authors hypothesize that intrinsic biological differences in the gut may help explain the different population-level vector transmission patterns. With regards to the general proteomics analysis, overall the authors highlight a more active midgut physiological/immune profile in low-incidence area MAN- versus high-incidence area RPE-derived sand flies, as per the detection of different enriched proteins in the former, such as PEPT1, PATH, a Na^+^/H^+^ exchanger (SLC9B2-like), paramyosin, SARA, XPR1, cytochrome P450, and CysPc, with recognized roles in gut motility, nutrient transport, immune modulation, and detoxification. Additionally, with regards to metaproteomics, while the profiles translated into a suggestive overall similar relative abundance at the phyla and class levels in both MAN and RPE populations (with the exception of Betaproteobacteria, with a lower relative abundance in the former), they authors also detected proteins referring to microorganisms identified uniquely in either RPE- or MAN-captured sand flies (16, and 36, respectively). They further mention that the signatures from some bacteria exclusively identified in the sand flies captured in the low-incidence area MAN, such as *Raoultella terrigena* and *Shewanella* sp., may be responsible for a potential *Leishmania*-refractoriness phenotype, as both microorganisms were previously described to have parasiticidal properties. We cannot exclude, though, that, on the other hand, unique bacteria detected in RPE-derived sand flies are responsible for their higher permissiveness to infection. Studies such as this add valuable insight to the leishmaniasis field (there are only a handful of studies available comparing two sites with different transmission dynamics) and can, e.g., lead to the identification of new bacteria that impact *Leishmania* development inside the vector midgut, to complement other already proposed refractoriness-promoting agents [21,22]. However, *Leishmania* transmission is multifactorial, and small but important ecological differences between sites (e.g., feeding and sugar sources, presence/absence of reservoir hosts…) may also contribute to the reported different transmission dynamics, something that needs to be clarified.

The second study investigates the vector competence of wild-caught *Nyssomyia antunesi* sand flies, collected from an urban park in Brazil, to two medically important parasites endemic to the Amazon region, *Leishmania* (*Viannia*) *lainsoni* and *Leishmania* (*Viannia*) *lindenbergi*, using as control laboratory-reared *Lutzomyia longipalpis* sand flies [20], a permissive vector capable of transmitting multiple *Leishmania* species [23,24]. Sánchez Uzcátegui et al. suggest that *Ny. antunesi* support the development of “at least a small population of late-stage promastigotes” of both *Leishmania* species and, thus, may be permissive to both [20]. However, although multiple parasite developmental stages were identified within the midgut of *Ny. antunesi* sand flies, including the infectious metacyclic promastigotes, in this species, parasites were present in lower numbers and located just posterior to the stomodeal valve, whereas in permissive *Lu. longipalpis* sand flies, they accumulated at the stomodeal valve. These differences may be critical for sand fly transmission, which depends on both the blockage of the anterior midgut by dense proteophosphoglycan and parasite masses, and on the damage of this valve, probably due to parasite adhesion, consequently leading to the regurgitation of the “infectious inoculum” into the host skin during blood feeding [25]. The authors suggest that these *Leishmania* species need more time to advance to the valve under laboratory conditions, as has been proposed for other parasite–sand fly pairings. However, this needs to be verified, together with the absolute and relative quantification of parasite forms, through transmission experiments. A larger sample size (a major limitation of the study is the relatively small number of wild-caught flies that developed infections) and experiments involving subsequent blood meals [26,27] are also necessary to prove the competence of *Ny. antunesi* sand flies to *L. lainsoni* and/or *L. lindenbergi* parasites. Notably, *Ny. antunesi* sand flies naturally infected with either of these *Leishmania* species have not yet been found in the wild. For this reason, field-based studies are also indispensable, as integrating laboratory infection experiments with entomological surveillance, molecular detection of parasites in wild populations, and ecological analyses is crucial to determine whether a sand fly species contributes to *Leishmania* transmission in endemic and urbanized landscapes, to accurately map transmission risk, and to inform the implementation of adequate control strategies in endemic regions.

The final two studies focus on human infection, addressing both disease epidemiology and the host immune factors relevant to clinical outcomes [28,29]. The first study provides a clearer picture of the epidemiology of cutaneous leishmaniasis in Israel via a nationwide molecular diagnosis approach. The detection of over 4168 PCR-confirmed cases, from 2017 to 2022 across five geographically diverse centers, by Avni et al. reveals that passive surveillance systems have substantially underestimated the true disease burden [28]. For comparison, during the same period, official records listed slightly more than 1400 cases [30], around one-third of those reported by Avni et al. after molecular confirmation. This gap highlights the limitation of clinical and microscopy examination alone and emphasizes the added value of PCR-based surveillance for the diagnosis of leishmaniasis. Notably, this study also uncovers an etiological diversity that was previously overlooked. Besides confirming *Leishmania major* (84%) and *Leishmania tropica* (14%) as the most common species circulating in Israel, the authors also identify *L. infantum* (2.7%), mostly associated with cutaneous manifestations, but also with visceral disease. Furthermore, this study reports, for the first time, the detection of New World *Leishmania* species in Israel, mainly belonging to the *Leishmania braziliensis* complex. Although these cases represent a small proportion of the total, some were associated with both cutaneous and mucocutaneous manifestations, highlighting the clinical importance of accurate *Leishmania* identification at the species level. Collectively, these results suggest that leishmaniasis surveillance is incomplete, biased, and clinically deficient in the absence of species-level molecular diagnosis tools.

The second study is a case report that addresses a distinct but equally critical challenge: the long-term control of VL in patients coinfected with HIV. Through an extended follow-up of three individuals residing in an *L. infantum*-endemic area who were treated with liposomal amphotericin B and remained relapse-free for more than eight years, Monge-Maillo et al. show that sustained control of *Leishmania* is achievable, even under chronic immunosuppression [29], despite the parasite’s ability to modulate the HIV life cycle and to hinder immune recovery [31]. By integrating functional cellular analyses (including those based on lymphoproliferative and whole-blood assays following stimulation with soluble *Leishmania* antigen), cytokine and chemokine measurements, and conventional clinical hematology profiling, the authors reveal the limitations of relying solely on CD4^+^ thresholds (<200 cells/µL) to predict relapse risk [29]. Similarly to others [32], they further emphasize that neither HIV viral load nor antiretroviral adherence reliably predicts clinical outcomes for *Leishmania* infection. The authors argue that *Leishmania*-specific cellular responses are the best surrogate readout of protective Th1 immunity, with the ability to predict relapse risk and capture the evolving nature of host–parasite immune interactions. In this context, their observation suggests that effective protection does not require a large number of T cells; rather, a relatively small pool of functional memory T cells could be sufficient. From a clinical perspective, these findings support including *Leishmania*-specific cellular immune monitoring into the routine follow-up of patients with VL/HIV coinfection, particularly in endemic settings, where improved risk stratification and better-informed decisions in the context of secondary prophylaxis are urgently needed.

Overall, the main goal of our Special Issue of compiling studies with a broad focus on leishmaniasis, with emphasis on the vectors, the hosts/reservoirs, the *Leishmania* spp. parasites, and the complex interactions established between each other, has been accomplished. Collectively, the studies published in this Special Issue highlight the intrinsic complexity of the field, on the one hand, advancing our knowledge in key areas, while, on the other, implying that there is much we still need to understand. Continuing the study on vector–parasite–host/reservoir interactions in health and disease, together with the improvement of diagnosis, surveillance, and longitudinal monitoring approaches, is essential to translate biological insight into multifaceted, integrated strategies to strengthen global control efforts [33,34,35] and “eliminate” leishmaniasis as a public health concern.

## Data Availability

No new data were created or analyzed in this study. Data sharing is not applicable to this article.
